# Segregation of Brain Structural Networks Supports Spatio-Temporal Predictive Processing

**DOI:** 10.3389/fnhum.2018.00212

**Published:** 2018-05-24

**Authors:** Valentina Ciullo, Daniela Vecchio, Tommaso Gili, Gianfranco Spalletta, Federica Piras

**Affiliations:** ^1^Neuropsychiatry Laboratory, Department of Clinical and Behavioral Neurology, IRCCS Santa Lucia Foundation, Rome, Italy; ^2^Neurosciences, Psychology, Drug Research and Child Health (NEUROFARBA), University of Florence, Florence, Italy; ^3^Department of Psychology, Sapienza University of Rome, Rome, Italy; ^4^IMT School for Advanced Studies, Lucca, Italy; ^5^Menninger Department of Psychiatry and Behavioral Sciences, Baylor College of Medicine, Houston, TX, United States

**Keywords:** predictive timing, spatio-temporal predictive performance, structural connectivity, diffusion tensor imaging, complex network theory, insula, schizophrenia

## Abstract

The ability to generate probabilistic expectancies regarding when and where sensory stimuli will occur, is critical to derive timely and accurate inferences about updating contexts. However, the existence of specialized neural networks for inferring predictive relationships between events is still debated. Using graph theoretical analysis applied to structural connectivity data, we tested the extent of brain connectivity properties associated with spatio-temporal predictive performance across 29 healthy subjects. Participants detected visual targets appearing at one out of three locations after one out of three intervals; expectations about stimulus location (spatial condition) or onset (temporal condition) were induced by valid or invalid symbolic cues. Connectivity matrices and centrality/segregation measures, expressing the relative importance of, and the local interactions among specific cerebral areas respect to the behavior under investigation, were calculated from whole-brain tractography and cortico-subcortical parcellation.

**Results:** Response preparedness to cued stimuli relied on different structural connectivity networks for the temporal and spatial domains. Significant covariance was observed between centrality measures of regions within a subcortical-fronto-parietal-occipital network -comprising the left putamen, the right caudate nucleus, the left frontal operculum, the right inferior parietal cortex, the right paracentral lobule and the right superior occipital cortex-, and the ability to respond after a short cue-target delay suggesting that the local connectedness of such nodes plays a central role when the source of temporal expectation is explicit. When the potential for functional segregation was tested, we found highly clustered structural connectivity across the right superior, the left middle inferior frontal gyrus and the left caudate nucleus as related to explicit temporal orienting. Conversely, when the interaction between explicit and implicit temporal orienting processes was considered at the long interval, we found that explicit processes were related to centrality measures of the bilateral inferior parietal lobule. Degree centrality of the same region in the left hemisphere covaried with behavioral measures indexing the process of attentional re-orienting. These results represent a crucial step forward the ordinary predictive processing description, as we identified the patterns of connectivity characterizing the brain organization associated with the ability to generate and update temporal expectancies in case of contextual violations.

## Introduction

The brain is capable of allocating attention dynamically on the basis of expectations concerning events occurrence, and this ability is critical for goal-directed behavior in a changeable environment. Indeed, real world information is often dynamic and imprecise, and the ability to derive timely and accurate inferences about changing contexts is crucial, given that prospective action control bears the potential for a successful interaction with the environment.

Behavioral findings (Coull and Nobre, 1998; Coull et al., 2000, 2013, 2016; Davranche et al., 2011; Carvalho et al., 2016) evidenced faster reaction times (RTs) to target stimuli preceded by informative pre-cues signaling event location and onset, indicating that attention can be deliberately and strategically oriented in space and time when overt (explicit) information is provided. Such behavioral optimization has been described as an endogenous process based on learned cue-target association that enhances action preparation (Coull and Nobre, 2008). Temporal expectations can also be derived more implicitly from the intrinsic predictive power of the flow of time, which is vector-like in nature, implying that probability of target occurrence increases with passing time (i.e., hazard function, Luce, 1986). Importantly, when prior expectations are violated, sensory (exogenous) information is integrated into the existing prediction model, and attention re-allocated to the novel context (Courville et al., 2006). For example, invalid trials with delayed target onset are detected faster than premature ones (Coull and Nobre, 1998), due to the increasing conditional probability over time that the event will occur given that it has not already occurred. Thus, both the explicit and the implicit nature of the source of temporal predictions modulate attentional orienting in order to optimize our interaction with unfolding sensory stimulation (Correa et al., 2005; Nobre et al., 2007; Correa, 2012).

Accordingly, computational approaches (Friston, 2005; Clark, 2013) have conceptualized the brain as an inference machine that integrates acquired experience about the world (prior believes) and incoming sensory information to generate probabilistic predictive models (posterior probabilities). Prior believes are updated in the light of new sensory input, allowing the system to arrive at posterior estimates. In other words, the posterior estimate depends on what is already known, and how much is learnt through the evidence, summarized in the probability of events occurrence (Hohwy, 2017). Any imbalance between expected and actual sensory data generates prediction error signals, computed as the difference between the prediction and the new evidence (Mathys et al., 2014), that adjust subjective expectations accordingly (Phillips et al., 2015; Hohwy, 2017). This theoretical framework is of particular clinical relevance, given that positive symptoms of schizophrenia (SZ) have been hypothesized as consequent to failures of predictive coding (Frith, 2005; Adams et al., 2013; Giersch et al., 2016; Sterzer et al., 2016; Martin et al., 2017), such that predictions are determined more by the new evidence and less by prior belief, while irrelevant stimuli are considered salient (e.g., delusion of reference) (Fletcher and Frith, 2009; Adams et al., 2013).

That’s why defining the neural basis of predictive coding is a major challenge in psychiatry and neuroscience. Conversely, functional Magnetic Resonance Imaging (fMRI) investigations on predictive timing in healthy subjects (Coull and Nobre, 1998; Coull et al., 2000, 2013, 2016; Davranche et al., 2011; Carvalho et al., 2016) have implicated complex and distributed networks -including the cerebellum, frontal cortex and inferior parietal cortex-, in forming temporal expectations, but the relevance of these structures in optimizing prospective motor behavior as a function of informative cues is still debated due to heterogeneity in experimental methods and the intrinsic complexity of the studied process (see Correa et al., 2006).

Pharmacological and fMRI evidence suggest that explicit and implicit processes of temporal expectations are functionally dissociable. Indeed, ketamine selectively impaired the ability to use internal estimates of time to make predictions (Coull et al., 2011), while partially distinct neuronal networks are preferentially activated both by the fixed temporal predictability of temporal cues and by the dynamic updating of temporal probabilities in the neutral cue condition (Coull et al., 2016).

However, as far as we know, the neural substrates of the attentional re-orienting in case of invalid trials and of the explicit forms of temporal expectations have been directly compared in one investigation only (Coull et al., 2000).

In recent years, advances in network neuroscience and graph theory proved to be effective tools in mapping neural connectomics (i.e., the pattern of neural elements and structural interactions of the neural system, Sporns et al., 2005), providing a deeper insight of the brain structural connectivity organization and its relation to behavior and cognition (Bullmore and Sporns, 2009; Rubinov and Sporns, 2010). Indeed, such network-based approach to MRI data analysis permits to quantify key structural properties and dynamics, which shape the anatomical substrate for functional specialized processing and information binding (Sporns et al., 2000, 2005).

Here we aimed at investigating the relationships between behavioral indices of explicit spatial and explicit-implicit temporal orienting effects and the brain structural network in a spatio-temporal predictive task in healthy subjects. To our knowledge, this is the first study that examined brain wiring in the context of explicit (i.e., based on learned cue-target association established through valid cueing) and implicit (i.e., derived from the sensory evidence in case of invalid cueing) predictive timing using centrality and segregation metrics derived from complex network analysis of diffusion tensor imaging (DTI) data. Specifically, we aimed at identifying the connectivity patterns underlying predictive behavior in terms of local connectedness (i.e., individual elements within the whole structural network having the highest number of connections) (Bullmore and Sporns, 2009) and local segregation (i.e., the number of connections that exist between the nearest neighbors of an element within the whole structural network) (Sporns et al., 2000) as to characterize the relative importance of, and the local interactions among specific cerebral areas respect to the behavior under investigation.

## Materials and Methods

### Participants

Twenty-nine healthy volunteers (age: 40.1 ± 12.3, 13F + 16M) were enrolled in the present study. Participants were recruited within the local community through advertising, and consecutively assessed at IRCCS Santa Lucia Foundation in Rome. All participants were screened for current or past diagnosis of any DSM-5 Axis I or II disorder using the SCID-5 Research Version edition (First et al., 2014) and SCID-5- PD (First et al., 2016a). Inclusion criteria were: (1) age between 18 and 65 years, (2) at least 8 years of education and (3) suitability for MRI scanning. Exclusion criteria included: (1) the presence of any psychiatric disorders according to DSM-5 criteria, (2) history of psychoactive substance dependence or abuse as investigated by the structured interview SCID-5-CV (First et al., 2016b), (3) history of neurologic illness or traumatic brain injury, (4) major medical illnesses, such as non-stabilized diabetes, obstructive pulmonary disease or asthma, hematological/oncological disorders, B12 or folate deficiency as indicated by blood concentrations below the lower normal limit, pernicious anemia, clinically significant and unstable active gastrointestinal, renal, hepatic, endocrine or cardiovascular system disease, newly treated hypothyroidism, (5) presence of any brain abnormality and microvascular lesion apparent on conventional FLAIR-scans (Iorio et al., 2013), (6) global cognitive dysfunction according to a Mini-Mental State Examination (MMSE) (Folstein et al., 1975) score lower than 24, consistent with normative data of the Italian population (Measso et al., 1993), (7) diagnosis of major neurocognitive disorder according to DSM-5 criteria and (8) non-Italian language native speaker.

All participants were right-handed, with normal or corrected-to-normal vision. They gave written informed consent to participate after the procedures had been fully explained. The study was approved and carried out in accordance with the guidelines of the IRCCS Santa Lucia Foundation Ethics Committee.

### Temporal and Spatial Orienting of Attention Task

Subjects were seated comfortably in a quiet room facing a Toshiba computer screen (1600^∗^1200 resolution 1280^∗^768 pixels, frame rate 60 Hz).

**FIGURE 1:**
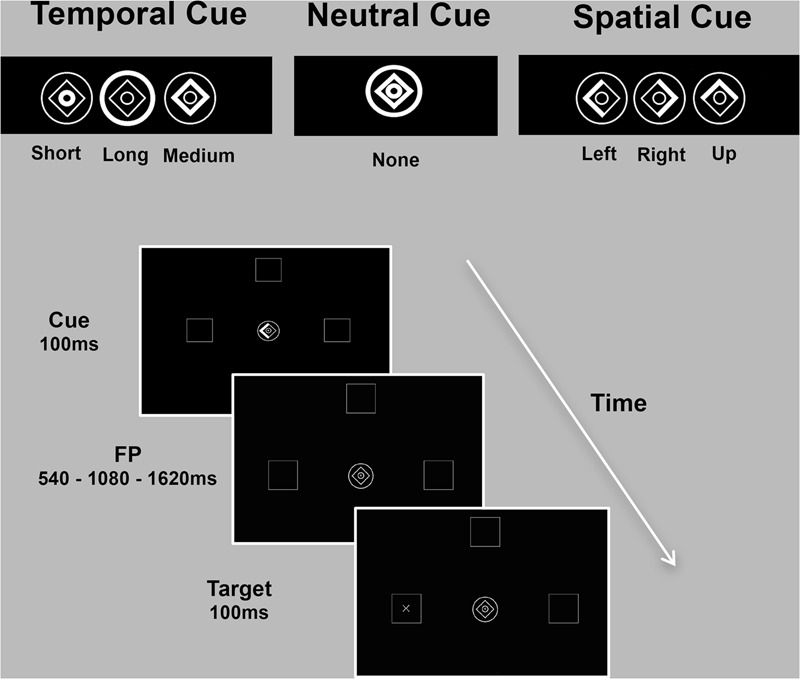
Spatio-temporal predictive task. Participants detected, as quickly as possible, visual targets (‘+’ or an ‘×’) appearing at one of three locations (left/up/right) after one of three intervals (short/medium/long). Expectations about stimulus location (spatial condition) or onset (temporal condition) were conveyed by symbolic cues with 80% validity. The cue consisted of a central image (1° eccentricity) composed of a diamond and two rings. Part of the cue briefly brightened (100 ms) to inform participants on the possible spatial location or temporal onset of the upcoming target. During the spatial condition, the left, up or right side of the diamond was highlighted to inform subjects that the target was likely to appear in the left, up or right peripheral box. In the temporal condition, brightening of the inner circle, diamond or the outer circle indicated that the target would occur after a short (540 ms), medium (1080 ms) or long (1620 ms) delay, respectively. During the neutral condition, no spatial or temporal information was provided. The inter-trial interval (ITI) varied randomly from 600 to 1000 ms. The three experimental conditions (spatial/temporal/neutral) were presented separately in three blocks of trials (90 each).

Predictive timing was investigated with an adaptation of the *temporal and spatial orienting of attention task* (Coull and Nobre, 1998), which measured RT to a briefly presented target (100 ms) appearing after one of three foreperiod durations (FP: 540 ms/1080 ms/1620 ms) in one of three boxes (left/up/right) depicted on the computer screen (7° eccentricity). Subjects were asked to press a response button to detect the target, as quickly as possible, using information provided by one of three types of cue. The cue was presented prior to the target and was either informative (spatial and temporal conditions) or uninformative (neutral condition). During the neutral condition, no spatial or temporal information was provided, as the whole image brightened. Spatial locations and temporal onsets were balanced across trials for all the three experimental conditions. In the majority of trials (80%) informative cues validly predicted where (spatial cue) or when (temporal cue) the target would appear (“valid” trials). In the remaining 20% of trials, the cue incorrectly predicted the spatial location or temporal onset of the target (“invalid” trials). The three experimental conditions (spatial/temporal/neutral) were presented separately in three blocks of trials and blocks’ order counterbalanced across subjects. The inter-trial interval (ITI) varied randomly from 600 to 1000 ms. A practice session (15 trials for the neutral and spatial condition each, 30 trials for the temporal condition) preceded the main task, which consisted of 90 trials for each experimental condition. In the temporal and spatial conditions, a total of 72 valid trials and 18 invalid trials were presented (**Figure 1**).

Stimuli presentation and collection of behavioral responses were controlled using e-Prime 2.0 software and a Serial Response Box Model 200a (Psychology Software Tools, Inc., Schneider et al., 2002), which allows for millisecond accuracy recording.

### MRI Acquisition

MRI data were collected at 3T (Philips Achieva) using a thirty-two channel receive-only head RF coil. A diffusion-weighted spin-echo echo-planar imaging sequence was used to acquire high angular resolution diffusion weighted images (HARDI) (Jones et al., 1999). One hundred twenty-eight gradient orientations and six unweighted (*b* = 0 s/mm2) images were acquired with the following parameters: TR/TE = 10000/76 ms, *b* = 1,000 s/mm^2^, 60 slices, slice thickness = 2 mm, FOV = 224 × 224, acquisition matrix = 112 × 112, resulting in data acquired with a 2 mm^3^ isotropic resolution. A high-resolution T1-weighted whole-brain structural scan was also acquired (1 mm^3^ isotropic).

### MRI Pre-processing

Data were preprocessed and analyzed in ExploreDTI v4.8.6 (Leemans et al., 2009). Data were corrected for motion and eddy currents. Motion artifacts and eddy current distortions were corrected with B-matrix rotation using the approach of Leemans and Jones (2009). During this processing procedure, all brain scans were rigidly normalized to Montreal Neurological Institute (MNI) space during the motion-distortion correction step.

### Tractography

Tensor estimation was performed using a non-linear least square method (Jones and Basser, 2004). Whole-brain tractography was performed using the DTI fiber tract-reconstructing algorithm implemented in ExploreDTI. The following tracking parameters were applied: step-size of 1 mm, minimum FA thresholds of 0.2 to initiate and continue tracking, an angle threshold of 30° and fiber length range 10–500 mm (Mori and van Zijl, 2002; Langen et al., 2012).

### Network Measures

The automated atlas labeling (AAL) atlas (Tzourio-Mazoyer et al., 2002) was registered to the HARDI data. A weighted connectivity matrix for each subject was generated in ExploreDTI using the AAL atlas regions as nodes. The atlas used to parcellate the gray matter consisted of 116 ROIs in total. However, the 26 cerebellar ROI were excluded for two main reasons: firstly, the cerebellum was not homogeneously covered by MRI across subjects, giving rise to a large inter-subject variability in the number of streamlines counted between cerebellar ROIS and the rest of the atlas; secondly, the streamline count from each cerebellar region resulted unavoidably altered by magnetic susceptibility artifacts due to the inhomogeneity of the local magnetic field induced by the magnetic field gradient used for diffusion tracking. Thus, a total of 88 nodes, 76 cortical and 12 sub-cortical, were used. The matrices used were weighted by number of streamlines connecting node i to node j. This matrix was thresholded to five streamlines and two nodes (i.e., left/right Heschl) were excluded from statistical analyses, in order to avoid singular matrices across subjects. Degree centrality and clustering coefficient (commonly used complex network measurements) were used to characterize network topology in terms of connectedness and segregation at the local level. All metrics were computed using the Brain Connectivity Toolbox (Rubinov and Sporns, 2010).

### Statistical Analysis

#### Behavioral Data

Reaction times to target stimuli faster than 100 ms were considered anticipatory and removed from the analysis. The benefit of temporal and spatial cueing on performance (i.e., individual mean RTs) was analyzed in one repeated measures ANOVA, with cue type (neutral, temporal valid, spatial valid) as the within-subjects factor. In order to examine the costs of invalid cueing and the potential interaction with the foreperiod length, another repeated measures ANOVA with cue type (time, space), validity (valid, invalid) and foreperiod (540, 1080, 1620 ms) as within-subjects factors was performed. A Bonferroni correction for multiple comparisons was applied. The benefits and costs of spatial cuing were explored by calculating 2 indices: (1) *Validity_spatial_* as the difference between RTs at spatial valid trials and RTs at neutral trials; (2) *Invalidity_spatial_* as the difference between RTs at spatial invalid trials and RTs at spatial valid trials. In order to further explore the temporal validity/invalidity effects unmasked by the foreperiod effect, 4 indices were calculated as follows: (1) *Temporal Validity_short_* as the difference between RTs at the short interval for early cue (V_540_) and RTs at the short interval when no cue was provided (N_540_); (2) *Temporal Validity_lon_*_g_ as the difference between RTs at the long delay for late cue (V_1620_) and RTs at the long delay when no cue was provided (N_1620_); (3) *Temporal Invalidity_short_* as the difference in RTs at the short interval for late cue (I_540_) and RTs at the short interval for early cue (V_540_); (4) *Temporal Invalidity_long_* as the difference between RTs at the long interval for early cue (I_1620_) and RTs at the long interval for late cue (V_1620_).

#### Neuroimaging Data

Behavioral indices (*Validity_spatial_, Invalidity_spatial_, Temporal Validity_short_, Temporal Validity_long_, Temporal Invalidity_short_, Temporal Invalidity_long_)* and network topology measures from the eighty-eight selected brain regions were included in the analyses, by investigating reciprocal dependencies. In order to look at relationships without inflating the risk of a type I error (of erroneously concluding that a significant correlation is present) (Draper and Smith, 1998), and to increase the number of descriptors in the regression equation as to improve predictors’ fit, a forward stepwise multiple regression model (*F* > 4 to enter) was chosen. Indeed, the forward stepwise procedure starts with no variables in the model and it tries out the variables one by one, including them if they are statistically significant, thus identifying the best set of predictors that gives the biggest improvement to the model. Simple linear regressions were preliminarily performed to include in subsequent multiple regressions analyses only variables significantly (*p* < 0.05) related to the behavioral measures considered.

All statistics were performed on StatView statistical software.

## Results

### Behavioral Data

A main effect of cue type (neutral, temporal valid, spatial valid) [*F*_(2,28)_ = 5.597; *p* = 0.0061] confirmed that targets appearing in a predictable location or moment in time are detected faster than uncued ones. *Post hoc* analyses with paired *t*-test evidenced a stronger effect for the spatial [*t*_(28)_ = 3.011; *p* = 0.0055] as opposed to temporal [*t*_(28)_ = 1.61; *p* = 0.1186] cueing, indicating that the latter did not significantly affect performance. The second analysis concerning temporal and spatial invalidity costs showed a significant effect of cue type, validity, and foreperiod [*F*_(1,28)_ = 23.71; *p* < 0.001; *F*_(1,28)_ = 89.3; *p <* 0.001; *F*_(2,56)_ = 75.83; *p* < 0.001 respectively], a significant cue × validity interaction [*F*_(1,28)_ = 32.23; *p <* 0.001], a significant validity × foreperiod interaction [*F*_(2,56)_ = 10.42; *p <* 0.001] and a significant three way interaction between cue type, validity and foreperiod [*F*_(2,56)_ = 4.10; *p =* 0.02]. *Post hoc* analyses (Bonferroni test) confirmed that for the temporal domain, RTs at the short interval after an early cue were not different from RTs for the medium interval at the medium cue, but significantly slower than RTs for the long interval at the late cue (due to the interaction with the foreperiod effect) [*p =* 0.0002]. Concurrently, RTs at the short interval for the late cue were slower from RTs at the medium interval after an invalid cue (either early or late) [*p <* 0.0001] and from RTs at the long interval after an early cue [*p <* 0.0001], while no difference was observed between the latter two variables. Behavioral performance at the medium interval was therefore discharged from the further indices computation and only RTs at the short and long delay considered (see **Table 1**).

**Table 1:** Mean reaction times (RTs) to target stimuli split by (a) cue type; (b) validity, and (c) the interaction between validity and foreperiod.

Variables	Means RTs	*SD*	Standard Error
**a) Cue type**			
Neutral cue	324.304	47.787	8.874
Time cue	318.027	51.604	9.583
Space cue	309.957	52.529	9.754
**b) Validity**			
*Validity_spatial_*	-14.345	25.661	4.765
*Invalidity_spatial_*	57.459	38.420	7.134
**c) Validity per foreperiod interaction**			
*Temporal Validity_Short_*	-14.932	38.378	7.123
*Temporal Validity_Long_*	-0.852	21.401	3.974
*Temporal Invalidity_Short_*	57.385	76.352	14.178
*Temporal Invalidity_Long_*	0.10	38.533	7.155

### Local Connectedness

#### Temporal Validity and Invalidity Effects

The covariance between the behavioral benefit of knowing in advance when the target will occur and the centrality of nodes within the whole structural connectivity network was investigated. Statistical analyses were separately performed for both *Temporal Validity_short_* and *Temporal Validity_long_* effects, considered as measures of explicit temporal orienting processes. Results from simple linear regressions showed significant negative correlations between the *Temporal Validity_short_* effect and degree centrality of a set of cortical regions. Moreover, a significant covariance between the degree centrality of a widespread pattern of cortical and subcortical nodes and the *Temporal Validity_long_* effect was found both as negative and positive correlations (see **Table 2**). Results from subsequent stepwise regressions showed that 51% of total variance observed in the *Temporal Validity_short_* effect was explained by right inferior parietal (*r* = -0.41), right superior occipital (*r* = -0.31) and left rolandic operculum (*r* = 0.48) nodes’ degree centrality variability [*F*_(3,25)_ = 10.854; *r =* 0.75; adjusted *R*^2^ = 0.51; *p* < 0.0001]. Further, for the *Temporal Validity_long_* effect the same analysis revealed that 67% of total variance was explained by right supramarginal (*r* = -0.29), left inferior parietal (*r* = -0.39), left fusiform (*r* = 0.51) and right insula (*r* = 0.24) nodes’ degree centrality variability [*F*_(4,24)_ = 15.455; *r =* 0.85; adjusted *R*^2^ = 0.67; *p* < 0.0001].

**Table 2:** Observed covariance between degree centrality and spatio-temporal predictive performance.

AAL Node	Node label	Partial coefficient	*R*	Adjusted *R*^2^	*F*	*p*
**Degree coefficient**						
*Temporal Validity_Short_*						
– Simple Linear Regressions						
17	Rolandic Operculum L		-0.56	0.29	12.364	0.0016
24	Frontal Superior Medial R		-0.40	0.13	5.093	0.0323
30	Insula R		-0.41	0.13	5.363	0.0284
50	Occipital Superior R		-0.39	0.12	4.727	0.0386
58	Postcentral R		-0.43	0.16	6.21	0.0191
62	Parietal Inferior R		-0.43	0.16	6.242	0.0189
89	Temporal Inferior L		-0.43	0.15	5.998	0.0211
– Stepwise Regression						
17	Rolandic Operculum L	-0.48	0.75	0.514	10.854	<0.0001
50	Occipital Superior R	-0.31				
62	Parietal Inferior R	-0.41				
						
*Temporal Validity_Long_*						
– Simple Linear Regressions						
1	Precentral L		0.37	0.10	4.264	0.0487
2	Precentral R		0.37	0.10	4.225	0.0496
23	Frontal Superior Medial L		0.42	0.14	5.744	0.0237
30	Insula R		0.41	0.14	5.425	0.0276
43	Calcarine L		0.43	0.15	6.068	0.0204
55	Fusiform L		0.66	0.41	20.881	<0.0001
61	Parietal Inferior L		-0.41	0.14	5.572	0.0257
64	Supramarginal R		-0.43	0.15	5.954	0.0215
68	Precuneus R		0.39	0.12	4.849	0.0364
71	Caudato L		0.48	0.20	8.091	0.0084
77	Thalamus L		0.38	0.11	4.542	0.0423
78	Thalamus R		0.41	0.14	5.578	0.0257
82	Temporal Superior R		0.42	0.14	5.636	0.0250
83	Temporal Pole Superior L		-0.41	0.14	5.555	0.0259
– Stepwise Regression						
30	Insula R	0.24	0.85	0.67	15.455	<0.0001
55	Fusiform L	0.51				
61	Parietal Inferior L	-0.39				
64	Supramarginal Gyrus R	-0.29				
*Temporal Invalidity_Short_*						
– Simple Linear Regressions						
38	Hippocampus R		0.43	0.16	6.262	0.0187
70	Paracentral Lobule R		0.39	0.12	4.78	0.0376
72	Caudate R		0.45	0.17	6.743	0.015
73	Putamen L		0.40	0.13	5.245	0.03
– Stepwise Regression						
70	Paracentral Lobule R	0.40	0.69	0.41	7.434	0.001
72	Caudate R	0.32				
73	Putamen L	0.44				
*Temporal Invalidity_Long_*						
– Simple Linear Regressions						
61	Parietal Inferior L		-0.44	0.17	6.608	0.016
88	Middle Temporal Pole R		0.40	0.13	5.096	0.0323
– Stepwise Regression						
61	Parietal Inferior L		-0.44	0.17	6.608	0.016
*Spatial Validity*						
– Simple Linear Regressions						
40	Para-Hippocampal R		0.53	0.26	10.724	0.0029
58	Postcentral R		-0.39	0.12	4.926	0.035
– Stepwise Regression						
40	Para-Hippocampal R	0.53	0.66	0.39	9.947	0.0006
58	Postcentral R	-0.39				
*Spatial Invalidity*						
– Simple Linear Regressions						
55	Fusiform L		0.49	0.21	8.442	0.0072

The covariance between the behavioral cost of receiving incoherent information about the target temporal occurrence and the centrality of nodes within the whole structural connectivity network was also investigated. Again, in the temporal invalid condition, two different variables were analyzed as to explore implicit temporal orienting processes (*Temporal Invalidity_short_, Temporal Invalidity_long_*). Positive and negative correlations between the two indices and nodes centrality of a cortical-subcortical network (see **Table 2**) were found. Results from stepwise regressions showed that 40% of total variance observed in the *Temporal Invalidity_short_* effect was explained by left putamen (*r* = 0.44), right paracentral lobule (*r* = 0.4) and right caudate nucleus (*r* = 0.32) nodes’ degree centrality variability [*F*_(3,25)_ = 7.434; *r =* 0.7; adjusted *R*^2^ = 0.4; *p* = 0.001]. Conversely, in the case of the *Temporal Invalidity_long_* effect, results from the stepwise regression evidenced a negative relationship between the ability to re-orient attention to a later time point and degree centrality of the left inferior parietal lobe [*F*_(1,27)_ = 6.608; *r =* 0.44; adjusted *R*^2^ = 0.2; *p* = 0.02].

#### Spatial Validity and Invalidity Effects

The analyses applied to subjects’ ability to orient attention in space evidenced that centrality of two regions was positively and negatively correlated with RTs to stimuli appearing in a predictable location. Results from the stepwise regression evidenced that the observed positive covariance between the *Validity_spatial_* effect and degree centrality of the para-hippocampal gyrus (*r* = 0.5) and the negative one with centrality of the post-central gyrus (*r* = -0.4) [*F*_(2,26)_ = 9.947; *r =* 0.7; adjusted *R*^2^ = 0.4; *p* = 0.0006] could explain 40% of the total covariance.

**FIGURE 2:**
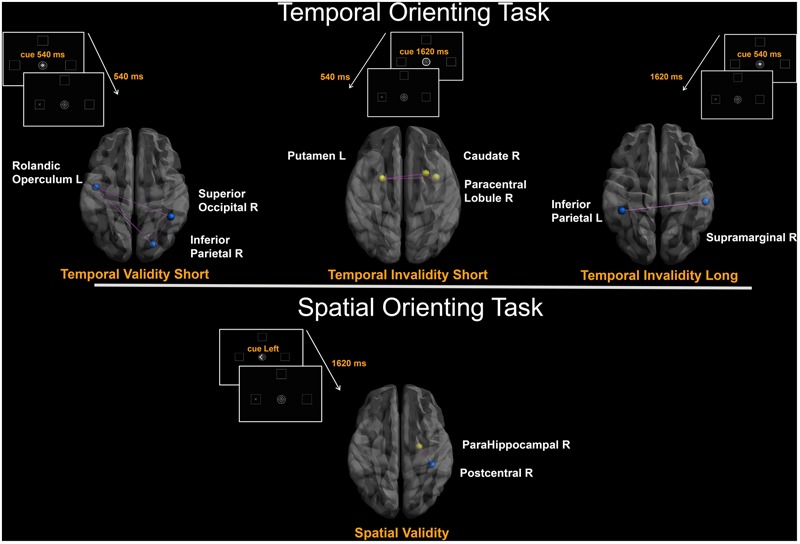
Degree-based connectivity networks for spatio-temporal predictive performance. Brain areas’ local connectedness associated with subjects’ performance according to: (a) *Temporal Validity_shor_*_t_; (b) *Temporal Invalidity_short_*; (c) *Temporal Invalidity_long_*_;_ (d) *Validity_spatial_* indices. Nodes size represents the mean number of streamlines incident to each area; link size represents the *R*^2^ value from the stepwise regression analyses. Blue = Negative correlation between performance and connectivity; Yellow = Positive correlation between performance and connectivity; Purple edges were drawn to represent links among areas.

The cost of spatial invalidity on performance (*Invalidity_spatial_)* was found to positively covary with degree centrality of one region. However, the stepwise regression model produced no significant result (**Table 2** and **Figure 2**).

### Local Segregation

#### Temporal Validity and Invalidity Effects

The covariance between the four temporal indices (*Temporal Validity_short_, Temporal Validity_long_, Temporal Invalidity_short_, Temporal Invalidity_long_*) and the prevalence of clustered connectivity around individual nodes was investigated. Simple linear regressions results showed significant negative correlations between the *Temporal Validity_short_* effect and clustering coefficient of nodes within a cortical network (see **Table 3**). Conversely, significant positive correlations were found between the *Temporal Validity_long_* effect and clustering coefficient of nodes within a cortical-subcortical network (see **Table 3**). The stepwise regression model showed a specific negative relationship between the *Temporal Validity_short_* effect and the clustering coefficient of the right superior frontal cortex, indicating that its’ connectedness with topological neighbors nodes explained 18% of the total variance [*F*_(1,27)_ = 7.001; *r = -*0.45; adjusted *R*^2^ = 0.18; *p* = 0.013]. Moreover, significant results from stepwise regression analyses indicates that 75% of total variance observed in the *Temporal Validity_long_* effect was explained by right putamen (*r* = 0.5), left paracentral lobule (*r* = 0.6) and right amygdala nodes’ clustering coefficient (*r* = 0.6) [*F*_(3,25)_ = 28.908; *r =* 0.9; adjusted *R*^2^ = 0.75; *p* < 0.0001]. The same analysis applied to the two temporal invalidity indices showed significant correlations between the latters and the clustering coefficient of a set of cortical nodes and in a single subcortical region (see **Table 3**). In the subsequent stepwise regressions, we found that the 50% of total covariance between the whole sub-network’s clustering coefficient and RTs indexing the *Temporal Invalidity_short_* effect [*F*_(2,26)_ = 12.31; *r =* 0.7; adjusted *R*^2^ = 0.5; *p* = 0.0002] was explained by the left middle frontal gyrus (*r* = 0.6) and the left caudate nucleus (*r* = 0.4). Conversely, when temporal expectations had to be updated to a later time point (as expressed by the *Temporal Invalidity_long_* effect), the clustering coefficient of the right posterior cingulate gyrus (*r* = -0.4), the right middle temporal pole (*r* = -0.4) and the left superior temporal pole (*r* = 0.4) explained the 40% of the observed covariance [*F*_(3,25)_ = 8.339; *r =* 0.7; adjusted *R*^2^ = 0.4; *p* = 0.0005].

**Table 3:** Observed covariance between clustering coefficient and spatio-temporal predictive performance.

AAL node	Node label	Partial coefficient	*R*	Adjusted *R*^2^	*F*	*p*
**Clustering coefficient**						
*Temporal Validity_Short_*						
– Simple Linear Regressions						
6	Frontal Superior R		-0.45	0.18	7.001	0.0134
7	Middle Frontal L		-0.42	0.15	5.907	0.022
14	Frontal Inferior L		-0.43	0.16	6.179	0.0194
26	Orbitofrontal Medial R		-0.39	0.12	4.862	0.0362
49	Superior Occipital L		-0.38	0.11	4.554	0.0421
68	Precuneus R		-0.43	0.15	5.979	0.0213
– Stepwise Regression						
6	Frontal Superior R		-0.45	0.18	7.001	0.0134
*Temporal Validity_Long_*						
– Simple Linear Regressions						
31	Anterior Cingulate L		0.42	0.14	5.658	0.0247
36	Posterior Cingulate R		0.46	0.18	7.123	0.0127
37	Hippocampus L		0.39	0.12	4.771	0.0378
38	Hippocampus R		0.43	0.15	5.992	0.0212
41	Amygdala L		0.49	0.21	8.449	0.0072
42	Amygdala R		0.46	0.18	7.305	0.0117
69	Paracentral Lobule L		0.65	0.41	20.29	0.0001
74	Putamen R		0.53	0.26	10.638	0.003
82	Superior Temporal Pole R		0.41	0.13	5.326	0.0289
87	Middle Temporal Pole L		0.37	0.11	4.272	0.0485
– Stepwise Regression						
42	Amygdala R	0.58	0.88	0.75	28.908	<0.0001
69	Paracentral Lobule L	0.57				
74	Putamen R	0.50				
*Temporal Invalidity_Short_*						
– Simple Linear Regressions						
7	Middle Frontal Gyrus L		0.43	0.15	5.961	0.0215
51	Middle Occipital L		0.37	0.10	4.247	0.0491
71	Caudate L		0.56	0.29	12.437	0.0015
– Stepwise Regression						
7	Middle Frontal Gyrus L	0.55	0.70	0.45	12.31	0.0002
71	Caudate L	0.41				
*Temporal Invalidity_Long_*						
– Simple Linear Regressions						
15	Inferior Frontal Orbitalis L		0.37	0.11	4.315	0.0474
36	Posterior Cingulate R		-0.41	0.14	5.569	0.0258
83	Superior Temporal Pole L		0.42	0.15	5.779	0.0233
88	Middle Temporal Pole R		-0.45	0.17	6.697	0.0154
– Stepwise Regression						
36	Posterior Cingulate R	-0.43	0.71	0.44	8.339	0.0005
83	Superior Temporal Pole L	0.40				
88	Middle Temporal Pole R	-0.35				
*Spatial Validity*						
– Simple Linear Regressions						
4	Frontal Superior R	-0.386	0.149	0.12	4.729	0.0386
*Spatial Invalidity*						
– Simple Linear Regressions						
19	Supp Motor Area L		0.49	0.18	7.156	0.012
35	Cingulum Post L		0.40	0.12	4.81	0.037
69	Paracentral Lobule L		0.45	0.17	6.694	0.0154
74	Putamen R		0.40	0.13	5.242	0.0301
83	Temporal Pole Superior R		0.39	0.12	4.898	0.0355
– Stepwise Regression						
69	Paracentral Lobule L	0.44	0.59	0.30	7.124	0.0034
74	Putamen R	0.39				

#### Spatial Validity and Invalidity Effects

In case of predictable targets, the clustering coefficient of a single area was found to covary with the ability to orient attention in space (*Valid_spatial_* effect). However, such correlation was no longer significant in the stepwise regression model.

Conversely, results evidenced positive correlations between the *Invalid_spatial_* effect and the clustering coefficient of cortical and subcortical areas (see **Table 3**). Results from the stepwise regression evidenced that 30% of the total covariance between the whole sub-network’s clustering coefficient and the cueing cost for spatial invalid trials [*F*_(2,26)_ = 7.124; *r =* 0.6; adjusted *R*^2^ = 0.3; *p* = 0.0034] was explained by the left paracentral lobule’s (*r* = 0.4) and right putamen’s (*r* = 0.4) clustering coefficient (**Table 3**).

## Discussion

In the present study, we investigated the covariance between performance on a spatio-temporal predictive task and topological measures of complex brain networks. Degree centrality (i.e., a measure of local connectedness identifying critical network elements as those having the highest number of connections or degree) and clustering coefficient (i.e., a measure of local segregation indicating the presence of clustered connectivity around individual nodes) were computed (Barabaìsi, 2016) and correlated to behavioral indices reflecting temporal and spatial expectations. Specifically, we aimed at investigating critical network elements for explicit spatial and temporal orienting, and to examine potential variations for implicit temporal expectations. Indeed, given that temporal predictions can rely on different sources of relevant temporal information (Correa, 2012), such as explicit predictive cues and probabilistic information associated with the passage of time (hazard function and foreperiod effects), we disentangled explicit processes from more implicit measures reflecting the interaction between these two different types of temporal orienting effects.

To date, although relevant behavioral (MacKay and Juola, 2007; Weinbach et al., 2015; Laidlaw and Kingstone, 2017) and fMRI (Coull and Nobre, 1998; Coull et al., 2015; Peer et al., 2015) investigations examined the interrelation between predictive behavior and processing of spatial and temporal stimuli features, this is the first study that applied complex network analysis to structural connectivity data for studying the brain topological organization underlying spatial and temporal predictive processing.

Here we found that response preparedness to cued stimuli relies on different structural connectivity networks for the temporal and spatial domains. Specifically, while the use of temporal informative cues was found to covary with centrality and segregation measures within a distributed cortical-subcortical network, its spatial counterpart was related to clustered connectivity around few brain regions. Particularly, the behavioral advantage of being validly cued to a certain spatial location was found to covary with local connectedness (increased when RTs were faster after valid as opposed to neutral cues) of a region in the right parietal lobe (the postcentral gyrus) more anterior relative to previous fMRI reports (see Chica et al., 2013 for a review; Coull and Nobre, 1998; Corbetta and Shulman, 2002). Although this region is traditionally linked to somatosensory perception (including proprioception), some functional and lesion studies (Corbetta, 1998; Balslev et al., 2013) suggested that the postcentral gyrus participates in movement organization and anticipation processes by contributing to code the locus of attention. Since spatial attention can be defined as the selection of locations for perception and/or for action, the postcentral gyrus may modulate the visual processing stream toward a motor response by using gaze-direction signals that have a proprioceptive component (Balslev et al., 2013). Alternatively, the observed correlation may be related to the postcentral gyrus participation to one of the three distinct key subsystems of attention, namely the orienting network (Fan et al., 2005; Posner, 2008). Indeed, clustered connectivity was found around the left paracentral lobule when the behavioral cost of being invalidly cued to a target location was considered. Since both the postcentral and the paracentral gyri participate to the network responsible for directing attention to target stimuli, triggered by specific spatial cues (Petersen and Posner, 2012), the patterns of relations among these brain areas and their topological neighbors may sustain cued orienting of attention to spatial locations. The fact that increased connectivity was found in a local network community comprising the right putamen when spatial invalidly cued trials were considered, seems to further confirm the hypothesis that this region is one of the hubs of the intrinsic connectivity networks in resting brain involved in attentional processes (Xiao et al., 2016).

As for temporal orienting, significant covariance was observed between centrality measures (calculated according to the number of axonal bundles incident upon a node) of regions within a subcortical-fronto-parieto-occipital network -comprising the left putamen, the right caudate nucleus, the left frontal operculum, the right inferior parietal cortex, the right paracentral lobule and the right superior occipital cortex-, and the ability to respond after a short cue-target delay suggesting that the local connectedness of such nodes plays a central role when the source of temporal expectation is explicit (Correa, 2012). When local segregation was taken into account, we found highly clustered structural connectivity across the right superior frontal gyrus, the left middle inferior frontal gyrus and the left caudate nucleus as related to explicit temporal orienting.

Interestingly, different connectivity clusters were observed when the automatic shifting of attention (related to unexpected premature targets) was separated from the voluntary top–down control of attentional orienting (in case of valid cuing) since clustering coefficient of the left middle frontal gyrus and caudate nucleus covaried with the former process, while the latter was related to the right superior frontal gyrus clustering coefficient.

These findings expand prior results (Coull and Nobre, 1998; Davranche et al., 2011; Coull et al., 2013, 2016; Carvalho et al., 2016) by defining, across the entire brain structural network, the relative importance of specific brain regions in temporal orienting. They also indicate the presence in the whole brain structural network, of a group of regions whose reciprocal connectivity scaled with the increased preparedness in responding to fast upcoming pre-cued stimuli and was specific for the process under investigation (automatic attentional shift vs. voluntary attentional orienting). Indeed, the functionality of a node is defined by the pattern of its connections with other nodes in the network (Bullmore and Sporns, 2009), since centrality increases as the potential for communication from a region increases, while the segregation measure here used is indicative of lasting patterns of relations among brain areas for supporting specific cognitive processes (Rubinov and Sporns, 2010).

Although a large corpus of research has implicated complex and distributed networks -including prefrontal, premotor, parietal and insula cortices and comprising also the striatum (Triviño et al., 2010)-, in temporal orienting of attention, the relevance of these structures in optimizing prospective motor behavior as a function of informative cues is still debated. Our results suggest that the behavioral benefit of being validly cued to a short interval is subtended by the reciprocal connectivity within a fronto-parieto-occipital complex. Activation of the inferior parietal cortex has been widely observed in fMRI studies on temporal orienting (Coull and Nobre, 1998; Coull et al., 2000, 2013, 2016; Cotti et al., 2011; Davranche et al., 2011). Although a frontoparietal network has been implicated in temporal as well as spatial orienting, the inferior parietal cortex seems specifically related to predictive timing (Wiener et al., 2010), as it has been found active not only when participants had to use temporal cues to optimize motor responses, but also for enhancing perceptual discrimination (Davranche et al., 2011), irrespectively of the effector and the type of requested action (button press/ocular saccades) (Cotti et al., 2011), both in the visual and auditory modalities (Merchant et al., 2013; Bolger et al., 2014). In terms of the structural network here observed, results confirm a pivotal role of this area in implementing the response benefits of temporal prediction, which is consistent with the thesis that temporal expectations tune action planning by optimizing prospective motor behavior (Coull and Nobre, 1998). The fact that densely connected local clusters were structurally centered around the right superior frontal gyrus in explicit temporal orienting supports the assumption that the latter is a voluntary process that requires more evolved structures such as the frontal cortex, involved in the strategic and voluntary (top–down) regulation of behavior (Konishi et al., 2008; Triviño et al., 2010).

In case of premature targets -target appears at the short interval after a late cue- a bottom–up, automatic grabbing of attention has been previously described and linked to the activity of the posterior extrastriate visual cortex (Coull et al., 2000). Here we found that premature targets are related to the local connectedness of the left putamen, the right caudate and the right paracentral lobule. The involvement of the striatum in timing is well known, being described as a ‘core timer’ of a more distributed neural network underlying temporal processing in the subsecond and multisecond range (Meck et al., 2008). Putaminal activity has been traditionally associated with motor preparation and execution, and specifically with the internal generation of precisely timed movements (Rao et al., 1997). Indeed, previous studies (Filip et al., 2016) suggested that the putamen is engaged in the evaluation of success and precision of the undergoing prospective temporal analysis, and our result would underscore its function as a neural node in a network engaged in cases of breaches of temporal expectations.

When the interaction between explicit and implicit temporal orienting processes was considered at the long interval, we found that explicit processes (indexed by the net advantage of being validly cued when the foreperiod effect was partialled out) were related to centrality measures of the bilateral inferior parietal lobule. Degree centrality of the same region in the left hemisphere covaried with behavioral measures (RTs at the long interval for early cue minus RTs at the long interval for late cue) indexing the process of attentional re-orienting. These results confirm the key role of the left parietal cortex in instantiating the behavioral benefits of temporal predictability, whether temporal information is conveyed by explicit predictive cues or by the probabilistic information associated with the passage of time (Coull et al., 2016). Densely connected local clusters were structurally centered on the right middle temporal pole and the right posterior cingulum when the re-orienting effect was specifically considered. The supramodal involvement of the temporal lobe in time processing has been already demonstrated (Kanai et al., 2011; Filip et al., 2016), while the posterior cingulate cortex seems necessary for organizing flexible behavior in response to an ever-changing environment. Indeed, this region contributes to signaling environmental changes and, when necessary, to recombining variables into strategy-specific measures of Bayesian evidence that the environment has changed (Pearson et al., 2011). The fact that lasting patterns of relations among the posterior cingulate and its topological neighbors were observable when the process of attentional re-orienting to a later point in time occurred, suggests that this area is specifically involved in the dynamic updating of current expectancies as a function of the foreperiod.

The selectivity of our results, indicating segregated structural connectivity networks for temporal and non-temporal stimuli dimensions, but also for explicit and implicit temporal orienting provides empirical support to the notion that complex networks theory enhances behavioral driven neuroimaging data analysis of predictive timing. Indeed, actual evidence on the neural underpinnings of temporal orienting is currently restricted to task-based fMRI experiments in which the core of the process circuitry is blurred by the recruitment of additional areas related to ancillary cognitive/sensory processes. Conversely, a task-independent structural neuroimaging approach within a network-based modular framework provides quantitative information on anatomical brain connectivity, at both global and regional levels, thus unraveling the lasting patterns of relations among brain areas for supporting the process under investigation (Sporns, 2013; Mišić and Sporns, 2016). We also think that our findings have a translational impact since alterations in predictive timing have been evidenced in psychiatric disorders such as schizophrenia. As such, we believe that our results have the potential to better define the neural circuits involved in the pathophysiology of the illness, and to provide an explanatory framework for symptoms and clinical manifestations. Given that graph theory allows a quantitative analysis of the pattern of interconnections each area has with other areas belonging to the same or different systems (Mišić and Sporns, 2016), its application may provide new insight on the dysfunctional interplay between timing deficits, clinical symptoms and *connectopathy* in SZ. Indeed, neuroimaging investigations evidenced connectivity deficits in patients with SZ, with positron emission tomography (Andreasen et al., 1996; Mallet et al., 1998), fMRI (Fornito and Bullmore, 2015; Crossley et al., 2016; Nelson et al., 2017) and diffusion-tensor imaging techniques (see Kelly et al., 2017 for a meta-analysis), suggesting that SZ pathophysiology may be explained in terms of abnormal or disrupted integration of spatially distributed brain regions (disconnection hypothesis, Friston and Frith, 1995; Friston et al., 2016). Here we observed that centrality and segregation properties of the parietal and frontal cortices modulate explicit and implicit temporal orienting in healthy subjects. Intriguingly, these areas have been identified as putative hubs across the whole brain network (Iturria-Medina et al., 2008; Gong et al., 2009) [i.e., highly connected nodes that allow segregated functional systems to share information by means of neural interaction (Collin et al., 2016)]. Such pivotal nodes infrastructure has been found disproportional in SZ patients, with abnormal high clustering, anomalous modularity structure (Zalesky et al., 2011; van den Heuvel et al., 2013), and reductions of global efficiency in the overall network structure (Zhang et al., 2012). Moreover, this altered connectivity profile was observed in specific areas (Bassett et al., 2008; van den Heuvel et al., 2010; Wang et al., 2012; Kaplan et al., 2016) that partially overlap with the predictive timing network here described, and are responsible for the computation of prediction error signals to guide learning and updating of expectancies (Kaplan et al., 2016).

Before concluding, few limitations have to be acknowledged. First, the relatively small sample size might reduce the impact of our findings and rescale the assertions here discussed. However, we intended to provide preliminary evidence of the neural architecture subtending the ability to optimize prospective motor behavior as a function of informative cues. In fact, complex and distributed networks have been thus far implicated in predictive timing, while aberrant connectivity has been suggested to underlie failures in predictive coding (Kraepelin, 1904; Friston et al., 2016). In addition, it might be argued that the network measures chosen are restricted to a small-sized pool, however, we intended to exploit the straightforward characterization given by the used ones, in order to elicit the cerebral interconnectivity correlates of such a complex process as predictive timing. Moreover, although the small number of temporal invalid trials here used could be a potential limitation, we nevertheless observed the traditional behavioral costs of being invalidly cued in time, further confirming the strength of the temporal orienting effects here investigated, even with a relatively reduced data pool. Lastly, recent studies on attentional orienting suggested that cues in the temporal and spatial conditions from the seminal study by Coull and Nobre (1998) are hardly comparable as spatial arrow cues elicit both voluntary and involuntary attention shifts (Hommel et al., 2001; Olk, 2014), being over-learned symbols of direction (Ristic and Kingstone, 2012) as opposed to temporal ones. Future behavioral and neuroimaging studies should employ cues that differ strictly in terms of temporal and non-temporal properties, in order to minimize potential confounds on the type of attentional orienting engaged.

## Author Contributions

FP conceived the experimental design, supervised the data analysis, contributed to results interpretation, and wrote the paper. VC conceived the experimental design, collected the data, performed the behavioral data analysis, contributed to results interpretation, and wrote the paper. GS supervised the data analysis, contributed to results interpretation, and revised the paper. TG conceived the experimental design, performed the MRI data analysis, contributed to results interpretation, and wrote the paper. DV conceived the experimental design, collected the data, performed the MRI data analysis, contributed to results interpretation, and revised the paper.

## Conflict of Interest Statement

The authors declare that the research was conducted in the absence of any commercial or financial relationships that could be construed as a potential conflict of interest. The reviewer MC and handling Editor declared their shared affiliation.
